# Clinical Reasoning: Perspectives of Expert Clinicians on Reasoning Through Complex Clinical Cases

**DOI:** 10.7759/cureus.51696

**Published:** 2024-01-05

**Authors:** Joseph H Donroe, Emilie Egger, Sarita Soares, Andre N Sofair

**Affiliations:** 1 General Internal Medicine, Yale School of Medicine, New Haven, USA; 2 Social and Behavioral Sciences, Yale School of Public Health, New Haven, USA

**Keywords:** expert clinicians, diagonostic error, general internal medicine, clinical reasoning, clinical skills, medical education

## Abstract

Introduction

Clinical reasoning is a core skill for physicians; most doctors do not attain the level of expertise associated with that of an expert clinician (EC). The purpose of this study is to identify the clinical reasoning strategies ECs prioritize when reasoning through complex cases.

Methods

We interviewed 14 ECs and performed a thematic analysis to identify strategies ECs prioritize when reasoning through complex clinical cases. The authors chose ECs based on the recognition of clinical and teaching expertise by trainees and other faculty members (ECs within our institution) and institutional recognition of high achievement in medicine and medical education (ECs outside our institution). We used a semi-structured guide to interview each EC, then reviewed and coded the interview transcriptions. We developed themes based on agreements between all transcript reviewers.

Results

We interviewed 11 male and three female ECs, one from outside the study institution. Two (14%) ECs were primary care physicians, and the remaining were sub-specialists. The authors organized strategies for clinical reasoning through complex cases around four themes, which were as follows: (1) connecting clinical reasoning to patient context; (2) embracing uncertainty, then reducing it; (3) returning to the patient’s bedside; and (4) remaining humble to limit diagnostic errors.

Conclusion

Clinical reasoning is a core clinical skill of physicians, and this article describes clinical reasoning strategies prioritized by ECs for complex clinical cases. Recognition and integration of these strategies into medical training and clinical educator practice may facilitate the evolution of clinical reasoning skills and reduce diagnostic errors.

## Introduction

The Institute of Medicine’s report “Improving Diagnosis in Health Care” promotes the development of a systematic, organized cognitive pathway to optimize clinical reasoning to correctly diagnose and treat a patient in a timely manner [1]. Moreover, many articles describe the theoretical basis of clinical reasoning [2-5]. Nonetheless, expert clinical reasoning remains elusive for most clinicians [3]. Understanding expert performance, including expert reasoning, for the purpose of scaffolding learners toward becoming expert physicians is of interest to medical educators [6, 7] because it allows the identification of domains of performance that can be improved and can provide strategies for improvement through deliberate practice [7].

Expert clinicians (EC) are recognized for their aspirational clinical skills, among them clinical reasoning. Through a small number of qualitative studies, researchers have attempted to characterize the ECs’ perception of their attributes, identify pivotal behaviors from their early careers, and explore their diagnostic processes [8-10]. Factors contributing to excellent clinical reasoning include breadth of knowledge and experience, deliberate development and effective application of clinical skills, manipulation of illness scripts, and the integration of knowledge with the patient story throughout the diagnostic process [6, 9]. Identifying such attributes of superior performance in clinical reasoning is a necessary step in the expert-performance approach to medical education and clarifying the diagnostic process [7].

Much is known about the diagnostic process [2-4]. Evidence suggests that ECs rely heavily on hypothesis generation and verification and that the early generation of hypotheses may be dependent upon clinical experience and the ability to store and retrieve experiential information [3]. However, gaps exist in our understanding of the processes behind hypothesis generation and verification and how experts prioritize clinical reasoning strategies when approaching complex cases [11]. This study aims to identify the most important strategies used by ECs when reasoning through complex cases. We identify themes related to ECs' prioritization of clinical reasoning strategies, including how they deal with uncertainty, anchor their reasoning in the patient context, and limit their diagnostic error.

## Materials and methods

Study design

This study was conducted at a single site, Yale New Haven Hospital, New Haven, CT, using an exploratory qualitative approach. Our site is a large academic hospital. The ECs participated in this study as part of a broader educational program called “Master Clinician Day" to expose trainees to ECs, highlighting their bedside clinical skills and career development. The ECs participated in a half-day of structured activities that highlighted their approach to clinical reasoning, patient care, bedside teaching, and career path.

Data collection

From May 2012 through July 2018, we interviewed 14 ECs using a semi-structured guide (Appendix A). We used purposive sampling to identify eligible participants for the study using the criteria that they were physicians trained in internal medicine and renowned for their clinical expertise and outstanding reputations as educators. Internal medicine faculty and trainees nominated institution-affiliated participants based on those criteria. All but one participant were physicians at our institution and thus well known to the author group. We interviewed one EC from outside the study institution who had been invited by the Department of Internal Medicine during the study period as a guest lecturer due to his high achievement in medicine and medical education. We enrolled participants iteratively, continuing to interview new participants until we reached theoretical saturation [12].

All physician authors (JD, AS, and SS) conducted interviews, which were recorded and transcribed verbatim using a professional transcription service. The primary investigator (JD) compared the audio recordings with the transcriptions for accuracy. Interviews ranged from 30 to 90 minutes in length. The interview guide asked about the attributes of ECs, their personal development as expert clinicians, and their reflections on the development of clinical expertise.

Data analysis

We used thematic analysis to identify and describe core themes across the interviews [13]. This was done in several phases, as described by Nowell et al. [14]. Initially, each physician author reviewed two to three transcripts and inductively coded all data felt to inform the research aim. The authors then convened to discuss the transcripts, agree upon each code, and generate a coding structure to use when coding subsequent transcripts. This process repeated until we coded all transcripts and had a shared agreement on the code structure. JD initially organized codes into themes. Collectively, JD, AS, and SS reviewed, refined, and agreed upon the themes. We used NVivo®11 software (QSR International, Burlington, MA) to manage data. The physician authors who conducted the interviews and developed the codes and themes are all internists and medical educators, and thus the data are viewed through this lens.

Ethical considerations

The study was determined to be exempt from review by the Yale University Institutional Review Board. All ECs consented to participate.

## Results

We interviewed 11 male and three female ECs, one from outside the study institution. Two (14%) ECs were primary care physicians; the remaining were specialists from the fields of cardiology, geriatrics, pulmonology, gastroenterology, infectious diseases, nephrology, and hematology.

We found that consistent with prior literature on expert reasoning, ECs universally emphasized the importance of a hypothesis-driven approach to clinical reasoning. This refers to generating and refining diagnostic hypotheses based on patient data and knowledge of the likelihood of a particular set of underlying diagnoses [5, 15, 16]. Additionally, themes emerged highlighting processes that ECs prioritized in their clinical reasoning that were complimentary or additive to a hypothesis-driven approach. These included (1) connecting clinical reasoning to the patient context; (2) embracing uncertainty, then reducing it; (3) returning to the patient’s bedside; and (4) remaining humble to limit diagnostic errors.

Theme one: connecting clinical reasoning to the patient context

Grounding the diagnostic process with the patient’s chief complaint is only one part of forming a coherent diagnosis. Coherency refers to the soundness of the connection between the diagnosis and the underlying physiology, symptoms, and predisposing factors [16]. According to one EC, “It's very easy to take data and veer away from the chief complaint. I think that we tend to look at data independently of the clinical presentation and go astray… It's very, very important to continue to anchor into the clinical presentation.” Beyond the chief complaint, ECs emphasized the critical importance of seeking to understand the broader patient context as foundational to clinical reasoning. “I think (an EC is) someone who has patience, someone who has a balanced view, looks at data, looks at the patient, is able to take a number of different factors in and assimilates data with the patients' backgrounds and patients' goals", said one EC. For some ECs, this broader context referred to an understanding of a patient’s goals of care at the end of life. Consideration of a patient’s goals, however, goes beyond end-of-life care, includes priorities at any stage, and requires the effective integration of clinical data through the lens of understanding the patient's story. Integrating clinical reasoning with patient context is a fundamental aspect of the art of medicine and can shift the role of the EC from a diagnostician to a problem solver. “That’s the art: understanding what a patient really needs. Whether they are more concerned about the tumor or they’re more concerned about being at their daughter’s wedding, living long enough to be at their daughter’s wedding with that tumor; which is their number one priority?”, opines an EC. In typical discussions of clinical reasoning, the emphasis is often on arriving at the correct diagnosis, though our ECs highlight that the complexity of the case often lies in understanding the patient’s priorities and adjusting the diagnostic workup and management accordingly.

Theme two: embracing uncertainty, then reducing it

Expert clinicians are skilled at connecting patient stories to illness scripts, facilitating early hypothesis generation [3]. This is challenging when reasoning through complex clinical cases due to uncertainty and the tremendous amount of information that must be processed. Underlying the EC's adeptness at integrating stories and illness scripts may be the EC's comfort with uncertainty and ability to quickly organize the data. Embracing the challenge of diagnostic uncertainty was a consistent characteristic of the ECs. "What you’re presented with when you see a complex patient is all the noise... be very comfortable to walk through that noise, to hear the music, to really say that I can slowly make sense of the noise and give appropriate weight to different findings, and... to suddenly see a picture that is fuller", stated one EC. Embracing uncertainty, ECs move towards reconciling data points, assessing the elements that fit and those that do not, to delineate the aspects of the case that are understood from those that remain unclear. Thus, complex cases are boiled down to their simplest form. One EC stated, “And so I think what I find... is that you need to strive to make the incredibly complicated as simple as can be.” Simplifying the case requires the expertise to assign varying relevance to the multitude of potential diagnostic clues. “I think that the ability to identify what really matters in a case, in other words, you get a lot of facts, and some of them are irrelevant and some of them are pertinent,” expressed one EC. Within those facts, identifying the “anchor point” or “fulcrum” around which to organize the data was an important skill.

Theme three: returning to the bedside

The fulcrum in a complex case is often revealed through the history and physical exam, and it is not surprising that ECs emphasize the importance of foundational bedside skills. According to an EC, “Master clinicians should be able to elicit a useful history, do a revealing physical exam, and be able to use data in an efficient way to make an appropriate diagnosis.” Several ECs specifically addressed the notion that the physical exam has lost its value in modern clinical practice. “So I think a master clinician has to be a master examiner of patients and be able to read the patient to pursue an informed examination, and I think that makes the difference, tremendous difference... So where the errors are being made is that people say that the physical examination has lost its value. A bad physical exam has lost its value, but a good examination, I think, is irreplaceable”, opined one EC. These elements of the history and physical exam often become the fulcrum around which effective clinical reasoning can be organized.

While the diagnostic process is often depicted as occurring linearly, for example in studies that analyze reasoning through simulated cases [11], actual complex diagnostic reasoning involves re-consideration of the case and a return to the patient. One EC stated, “And 25 years later, I still feel exactly the same way. When people don’t get the answer right, my instinct is always to say that something was missing from history." Another EC said, “If what’s unclear in the case is the complaint and how it is related to what I understand, then I’ve got to go back and figure out what I’ve missed. Often, it’s that I didn’t get a historical feature.” An emphasis on the return to the patient’s bedside to confirm or obtain further elements of the history and physical exam is particularly important in the modern era where time spent with patients has become more limited [17,18]. 

Theme four: remaining humble to limit diagnostic errors

Techniques to reduce diagnostic errors and the dual process theory of reasoning are well described in the literature [5,19,20]. A recent review underscores the importance of clinical experience in the generation and refinement of diagnostic hypotheses using intuitive, non-analytic processes by experts [3]. Humility, while to some extent innate, is also honed through patient care experiences, and ECs in our study identified humility as important to effective clinical reasoning. For our purposes, the construct of humility has several key elements, including accurate self-assessment, the ability to acknowledge one’s mistakes and limitations, openness to new ideas and contradictory information, and keeping one’s abilities and accomplishments in perspective [21]. “I think that internal medicine is a very humbling field. I've been studying it for 40 years now, and I don't feel like I have mastery of it,” said one EC. A humble approach becomes an important check on clinical reasoning as it includes acceptance of one’s limitations and an openness to the possibility of being wrong. “I think one of the important attributes of a master clinician is the willingness to doubt yourself and to reconsider diagnoses”, opined another EC. Reflective thinking is an important part of humility and can identify one’s own tendency towards certain biases and heuristics that may lead to a diagnostic error [22, 23]. All ECs reflected on prior errors in judgment in patient care that shaped their current practice.

## Discussion

Expert clinicians are recognized for their teaching, patient care, clinical skills, and exceptional clinical reasoning. In this study, we identified strategies prioritized by ECs when reasoning through complex clinical cases through structured interviews with ECs from a range of internal medicine disciplines. These strategies include: 1) connecting clinical reasoning to the patient context; 2) becoming comfortable with uncertainty and then reconciling data points to simplify the case; 3) returning to the patient's bedside to clarify the history and perform an effective exam; and 4) remaining humble to limit diagnostic errors.

Prior studies into the experience of ECs have focused on development and practices that sustain clinical excellence [10, 24-27]. Expert reasoning has been primarily addressed in the literature through reviews of the cognitive psychology of reasoning or in perspective pieces, though some recent studies have attempted to better characterize the diagnostic approach of experts. Table 1 highlights selected expert clinical reasoning strategies [3, 4, 8, 28].

**Table 1:** Selected clinical reasoning strategies used by expert clinicians

Clinical reasoning strategy	Source
Expanding knowledge and experience	Mylopoulos et al., 2012 [6]
Mastering the clinical skills of history-taking and physical exam	Mylopoulos et al., 2012 [6]
Skillful manipulation of illness scripts	Kumar et al., 2021 [9]
Integrating clinical knowledge with the patient's story	Mylopoulos et al., 2012 [6]
Generating diagnostic hypotheses early in the encounter	Brush et al., 2017 [3]
Connecting clinical reasoning to the patient context	Donroe et al., Current publication
Embracing uncertainty, then reducing it by reconciling data	Donroe et al., Current publication
Returning to the patient's bedside	Donroe et al., Current publication
Remaining humble to limit diagnostic errors	Donroe et al., Current publication

Kumar et al. took a quantitative approach to investigate differences in diagnostic reasoning between ECs and their peers in internal medicine and identified that ECs are likely more facile in their use of illness scripts [9]. Our study concurs with the proficient use of illness scripts by ECs and offers a possible bridge to how ECs use illness scripts more proficiently. Specifically, our ECs highlighted the important skill of reconciling data points and simplifying the case down to the features with the most diagnostic weight. Mylopoulos et al. touched on aspects of clinical reasoning in their qualitative study of perspectives on expert diagnostic practice [6]. They identified four themes of expert diagnostic practice: 1) possession of extensive knowledge; 2) possession of skills to gather the patient story; 3) reflective integration of knowledge and patient stories; and 4) continuous learning through clinical practice [6]. Our study is consistent with many of their findings, specifically the importance of collecting data through an effective history and physical exam and the importance of the patient story. We expand on these findings to emphasize the role of the EC as a problem solver, grounding their reasoning not only in the chief concern but also in the broader context of the patient's goals and priorities. In the qualitative work of Wadhwa et al., clinicians recognized humility as important to improving clinical practice through developing expertise and providing better patient care [29]. Our study explicitly links humility to expert reasoning and suggests it as an attribute used by experts to reduce diagnostic errors. A potential mechanism was described by Trinh et al., where humility consistently moderated the negative effect of expertise on flexibility. In other words, experts with higher levels of humility were more likely to have flexible and adaptive approaches [30].

A goal of “Master Clinician Day” at our institution was to highlight the ECs’ clinical reasoning through a complex case so that trainees could advance their own clinical reasoning skills. Now, through an analysis of structured interviews with those ECs, we have identified and described several of the strategies they employ in their clinical reasoning. The educational value of elaborating on these strategies is considerable. Trainees have ample opportunity to practice clinical reasoning skills, and familiarity with these strategies can lead to more deliberate practice with effective strategies. Explicit role modeling and teaching of these strategies by clinical educators can be an important tool for helping trainees develop clinical reasoning skills. Finally, understanding the clinical reasoning strategies used by ECs provides trainees with standards by which to measure their own diagnostic approach.

Our study has some important limitations. Expert clinicians were selected based on a trainee or peer acknowledgment of their qualifications rather than using objective measures of their credentials as an EC, as such measures do not exist. Faculty and trainees nominated ECs based on their working experience with them, however, which may minimize the risk of including non-expert clinicians in the study. Additionally, ECs were primarily identified from a single academic center and from internal medicine-based specialties. Expert clinicians are present in other specialties, and those voices are not represented in this article. Finally, this study relied on the ECs’ self-analysis of their diagnostic process and thus may be prone to hindsight errors. However, our intent was to capture the internal reflections of ECs, and thus we specifically chose qualitative methods for this study.

## Conclusions

Clinical reasoning is a core clinical skill of physicians, and despite an emphasis on clinical reasoning during training, most physicians do not reach the clinical reasoning skill of an EC. In this article, we highlight clinical reasoning strategies prioritized by ECs for complex clinical cases. Recognition and integration of these strategies into medical training and clinician-educator practice may facilitate the evolution of clinical reasoning skills and reduce diagnostic errors. Increasing early physician exposure to ECs, role modeling clinical reasoning strategies used by ECs, and creating a culture of humility within medical training programs may be important strategies to improve clinical reasoning and merit further research.

## Appendices

Appendix A 

**Table 2:** The expert clinician interview guide

Interview Guide: Domains and Questions
Domain: General reflections on a master (expert) clinician
1. What are the attributes of a master (expert) clinician?
2. Who are some individuals that you would identify as master/expert clinicians? What characteristics do you think they share?
Domain: Personal development as a master (expert) clinician
1. Did you have a role model or mentor whom you considered to be formative in your educational process?
2. During your training (either in medical school, residency, or fellowship), were there any seminal events that encouraged you to strive for excellence in the clinical arena?
3. How does your involvement with students, residents, and fellows contribute to your achievement of clinical skills?
4. What were the elements outside of medicine that were most influential in your development as an expert clinician?
5. Reflecting back, can you recall any clinical mistakes that you have made or unexpected patient outcomes that resulted in important, formative lessons or changed the way you practice or approach medicine? What are those lessons?
Domain: Development of clinical expertise
1. Do you think that one can be taught to become a master (expert) clinician? If so, what are the steps towards this goal?
2. In your opinion, what are the barriers to becoming master (expert) clinicians that young physicians face?
3. What mistakes in clinical reasoning do you see most often made by young physicians?
4. One thing that seems to separate the expert clinician from others is their ability to reason through a complicated patient and connect the pieces of the history in a very clear way. Do you have a particular approach when faced with a patient whose presentation is complex and whose diagnosis is unknown?
